# Pain sensitivity in men who have never experienced a headache: an observer blinded case control study

**DOI:** 10.1186/s10194-021-01345-0

**Published:** 2021-11-08

**Authors:** Isa Amalie Olofsson, Jeppe Hvedstrup, Katrine Falkenberg, Mona Ameri Chalmer, Henrik Winther Schytz, Miguel Benjamin Pedersen, Henrik Ullum, Ole Birger Pedersen, Jes Olesen, Thomas Folkmann Hansen

**Affiliations:** 1 Department of Neurology, Danish Headache Center, Copenhagen University Hospital, Valdemar Hansens Vej 5, Glostrup, Denmark; 2 Statens Serum Institut, Copenhagen, Denmark; 3 Department of Clinical Immunology, Zealand University Hospital, Koege, Denmark

**Keywords:** Never headache, Quantitative sensory testing, Total tenderness score, Cold pressor test, Pain threshold, Pain sensitivity, Never had headache

## Abstract

**Background:**

Headache affects 90–99% of the population. Based on the question “Do you think that you never ever in your whole life have had a headache?” 4% of the population say that they have never experienced a headache. The rarity of never having had a headache suggests that distinct biological and environmental factors may be at play. We hypothesized that people who have never experienced a headache had a lower general pain sensitivity than controls.

**Methods:**

We included 99 male participants, 47 headache free participants and 52 controls, in an observer blinded nested case-control study. We investigated cold pain threshold and heat pain threshold using a standardized quantitative sensory testing protocol, pericranial tenderness with total tenderness score and pain tolerance with the cold pressor test. Differences between the two groups were assessed with the unpaired Student’s t-test or Mann-Whitney U test as appropriate.

**Results:**

There was no difference in age, weight or mean arterial pressure between headache free participants and controls. We found no difference in pain detection threshold, pericranial tenderness or pain tolerance between headache free participants and controls.

**Conclusion:**

Our study clearly shows that freedom from headache is not caused by a lower general pain sensitivity. The results support the hypothesis that headache is caused by specific mechanisms, which are present in the primary headache disorders, rather than by a decreased general sensitivity to painful stimuli.

**Trial registration:**

Registered at ClinicalTrials.gov (NCT04217616), 3rd January 2020, retrospectively registered.

## Introduction

Headache is an extremely common illness affecting 90–99% of the population [1–3]. The primary headache disorders, migraine and tension type headache, have lifetime prevalences of 16–25% and 78–89%, respectively [1, 4, 5]. Based on the question “Do you think that you never ever in your whole life have had a headache?”, 4% of the population report that they are free from headache [1, 6]. The rarity of never having had a headache suggests that distinct biological or environmental factors may be at play. We previously described a group of headache free people regarding several socio-demographic factors, but found only small differences compared with a matched control group [6].

The reason why some people never experience headaches could be explained by a generally lower pain sensitivity. This is supported by previous studies in primary headache disorders. In tension type headache and migraine a lower pain detection threshold compared with healthy controls have been reported, however some studies have shown negative or conflicting results [7–18]. Pericranial muscle tenderness is increased in tension type headache and in people with migraine [19–22]. Studies with experimental pain using the cold pressor test showed a lower pain tolerance in chronic tension type headache and conflicting results in migraine [23–27].

In the present study, we investigated whether pain sensitivity differed between men who have never experienced a headache and controls. We hypothesized, that people who have never experienced a headache had a higher pain threshold to cold and heat by quantitative sensory testing, a lower pericranial muscle tenderness by total tenderness score and a higher pain tolerance by cold pressor test. We tested this in an observer blinded nested case-control study.

## Material and methods

### Study population

**Fig. 1:**
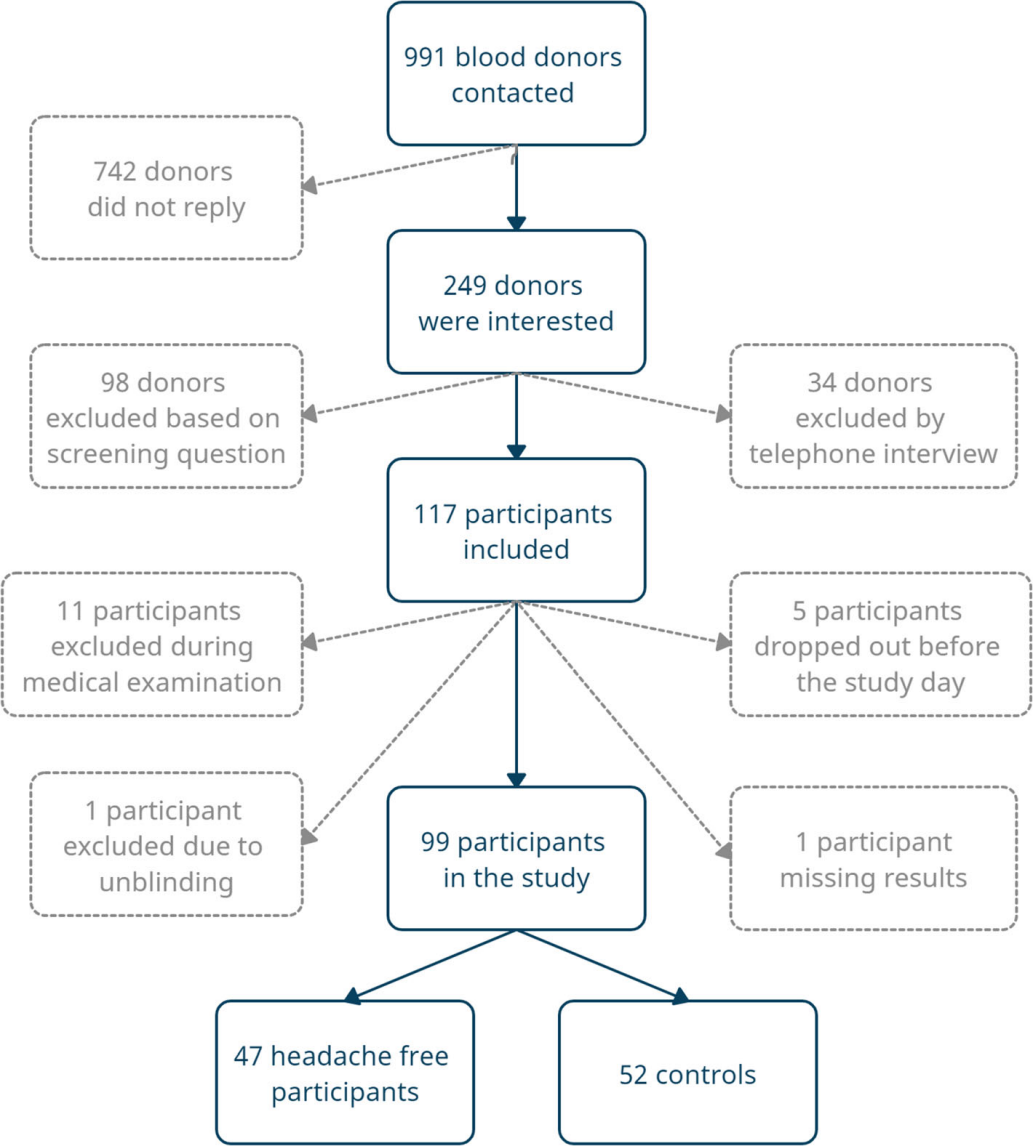
Inclusion of participants. Flowchart illustrating inclusion and exclusion of participants for the study

We recruited 100 male participants from the Danish Blood Donor Study (DBDS). The DBDS is an ongoing national cohort study with voluntary participation of more than 137,000 Danish blood donors [28]. The blood donors are largely representative of the whole Danish population [29]. Seventeen thousand four hundred thirty-four male blood donors had answered the question “Do you think that you never ever in your whole life have had a headache?” in a digital tablet-based questionnaire [30]. Based on the question possible participants were identified and a sample were drawn using simple random sampling by means of encrypted ids. Through secured electronic mail (e-Boks), we invited 991 eligible participants to the study [31]. Six hundred fifty male participants who had never experienced a headache and 341 randomly selected male participants. Flowchart of inclusion of participants, see Fig. 1. Participants filled out a digital questionnaire with contact information and consented to a telephone interview regarding the study. All participants answered the question “Do you think that you never ever in your whole life have had a headache?” again in the digital questionnaire, to minimize the risk of misclassification of case-control status. Two hundred forty-nine participants filled out the questionnaire. Ninety-eight participants were excluded based on the screening question. Inclusion criteria were: Male sex, 18–70 years of age and a weight of 45–95 kg. Exclusion criteria were: Serious somatic or psychiatric disease or daily medication use. One hundred fifty-one possible participants were contacted by telephone to make sure they met inclusion criteria and not the exclusion criteria. One hundred seventeen participants were included and invited for further screening at the Danish Headache Center, Rigshospitalet. Five participants dropped out before the study day. One hundred twelve participants were interviewed and underwent a medical examination with diagnostic interview for primary headache disorders by trained senior medical students, supervised by Professor Jes Olesen. Eleven participants were excluded after the medical examination and one participant was excluded due to unblinding on the study day. One hundred participants completed the study, but data were missing for one participant, giving a total of 99 study participants, 47 headache free participants and 52 controls, for data analysis. The study was performed between October 2019 to March 2020. Participants received a 500 DKK (67 EUR) reimbursement for participation in the study.

### Design

This study was an observer blinded nested case-control study. Forty-seven headache free male participants and 52 randomly selected male controls completed the study. All participants were examined by the same observer (IAO) who was blinded to the case-control status of the participants. The case-control status remained blinded until after data management. Blinding was performed by study investigator (TFH), who was not involved in data collection or data analyses.

### Ethical approval

All participants gave oral and written informed consent prior to any study-specific procedures. The study was approved by the Ethics Committee of Copenhagen (H-19022744) and the Danish Data Protection Agency. The study was registered at ClinicalTrials.gov (NCT04217616) and was conducted according to the Declaration of Helsinki of 1964, 2013 version [32].

### Study procedure

All participants arrived non-fasting at the Danish Headache Center, Rigshospitalet between 8.00 and 13.15. Participants were without any kind of headache at least 48 h before the study day and without any medication use at least 12 h before the study day. The participants were instructed not to talk about their medical history, family medical history or headache with the observer. One participant was excluded due to unblinding of the case-control status and was replaced by inclusion of another participant. All participants were examined by the same observer (IAO). The participants stayed in the examination room for 30 min before testing to acclimatize. Pain perception was examined using standard quantitative sensory testing, total tenderness score and cold pressor test.

### Quantitative sensory testing

The standardized quantitative sensory testing (QST) protocol from the German Research Network on Neuropathic Pain (DFNS) includes measurement of 13 parameters [33, 34]. We selected cold pain threshold (CPT) and heat pain threshold (HPT). All participants were tested according to the QST protocol using the TSA-II thermode (Medoc, Ramat Yishai, Israel). The test sites were the dorsal side of the non-dominant hand (radial nerve innervated) and the corresponding side of the forehead (V1 of the trigeminal nerve, innervated). All tests were illustrated and practiced on the dominant hand, before the actual measurements were taken. The thermode had a contact area of 7.84 cm2 and was attached to the skin with constant pressure by a strap. The baseline temperature of the thermode was 32 °C and threshold was obtained with ramped stimuli (1 °C /second) that were terminated when the participant pressed a button. The participants were instructed to press the button when the cold/warm sensation first became painful. Cut-off temperatures were 0 and 50 °C. Inter-trial-time was a least 12 s. During the experiment, the participants were not able to watch the computer screen. The first measurement was discarded, and the mean threshold temperature of the next three consecutive measurements was calculated.

### Pericranial tenderness

Pericranial muscle tenderness was evaluated using the total tenderness scoring system [35, 36]. Eight paired pericranial muscles and tendons were manually palpated in a standardized manner with small rotating movements for 4–5 s (m. masseter, m. pterygoideus lateralis, m. temporalis, m. frontalis, mastoid processes, m. sternocleidomastoideus, occipital muscle insertions and m. trapezius). The participants were asked to score tenderness on a 4-point scale (0–3). 0 = no visible reaction or verbal report of tenderness, 1 = no visible reaction but verbal report of tenderness or mild pain, 2 = verbal report of painful tenderness, facial expression of discomfort or no reaction, 3 = marked grimacing or withdrawal, verbal report of marked painful tenderness and pain. All participants were tested by the same observer (IAO) who was trained to exert a pressure of 140 arbitrary units (80 kPa). Training was performed with a palpometer [36, 37]. The score from each location was added to get a total tenderness score (TTS) ranging from 0 to 48. The TTS was divided into a cephalic tenderness score according to sensory innervation by the trigeminal nerve (m. masseter, m. pterygoideus lateralis, m. temporalis and m. frontalis) and a neck tenderness score according to sensory innervation by the upper cervical roots (mastoid processes, m. sternocleidomastoideus, occipital muscle insertions and m. trapezius) [20].

### Cold pressor test

The cold pressor test was performed by having the participants submerge their non-dominant hand up to the wrist in ice water. 3 L of room temperature water and 2 L of ice were mixed in a bucked. The water was prepared 1 min prior to testing. The participants were asked to submerge the hand and to keep it in the water for as long as they could. Participants were made aware that they could move the hand or keep it still as they wished. The maximum possible tolerance time was limited by the observer to 10 min, at which point the participants were asked to remove the hand from the water. The participants were not informed of the time limit. Tolerance time was recorded, and participants rated the intensity of the pain on a verbal rating scale (VRS) from 0 to 10 (0 = “no pain”, 10 = “the worst pain possible”).

### Statistical analysis

Normally distributed data were presented as mean values with 95% confidence intervals and non-normally distributed data as median values with interquartile range. Differences between the two groups were assessed with the unpaired Student’s t-test or Mann-Whitney U test as appropriate. Data for tenderness scores were logarithmically transformed to achieve a normal distribution. Primary outcomes were difference in median cold pain threshold, median heat pain threshold and mean total tenderness score between the two groups. Secondary outcomes were difference in median pain tolerance time and mean pain score on the VRS during the cold pressor test. As 60 participants (59.4%) endured the entire cold pressor test of 600 s, post hoc analyses were performed on pain tolerance time using survival statistics with Kaplan-Meier curves and a log rank test. Baseline data on weight were missing for 2 participants and mean arterial pressure were missing for 3 participants. The remaining variables had complete data on all 99 participants. Calculation of sample size was based on the assumption that headache free participants would have a mean total tenderness score of 5 and controls would have a mean total tenderness score of 7. With a standard deviation of 3, a significance of 0.05 and a power of 0.80 at least 36 participants in each group should complete the study. As there was no literature on cold or heat pain threshold in headache free participants, we included additional participants to strengthen our statistical power in order to detect a difference between the groups. All analyses were performed using R statistical software (version 4.1.0) and R studio (version 1.2.5001), [38]. The level of significance was set at 5%.

## Results

In total, 99 male participants completed the study, 47 headache free participants and 52 controls. Headache free participants had a mean age of 52.9 years (95% CI 48.7–57.1) and controls had a mean age of 51.7 years (95% CI 48.1–55.3). There was no significant difference in age between the two groups (*P* = 0.66). There was no difference in mean weight between headache free participants, 81.9 kg (95% CI 79.2–84.6 kg), and controls, 82.1 kg (95% CI 79.8–84.4 kg), (*P* = 0.91). Headache free participants had a mean arterial pressure (MAP) of 106.8 mmHg (95% CI 103.6–109.9 mmHg) and controls had a MAP of 103.6 mmHg (95% CI 100.9–106.3 mmHg). There was no difference in MAP between headache free participants and controls (*P* = 0.13).

Of the 52 controls, 21 (40%) had a primary headache disorder; 5 (10%) controls had episodic migraine, 1 (2%) control had frequent episodic tension type headache and 15 (29%) had infrequent episodic tension type headache. The remaining 31 controls had a history of headache but did not fulfill diagnostic criteria for a primary headache disorder at the time of inclusion in the study.

### Quantitative sensory testing

There was no difference in any of the QST variables between the two groups (Table 1). Median cold pain threshold (CPT) on the hand was 1.1 °C (IQR 0.0–9.0 °C) in headache free participants and 1.9 °C (IQR 0.0–7.2 °C) in controls, (*P* = 0.81). Median CPT on the forehead was 4.6 °C (IQR 0.0–8.4 °C) in headache free participants and 5.05 °C (IQR 0.9–9.7 °C) in controls, (*P* = 0.38). Median heat pain threshold (HPT) on the hand was 49.1 °C (46.8–50.0 C°) in headache free participants and 48.8 °C (46.8–50.0) in controls, (*P* = 0.49). Median HPT on the forehead was 48.3 °C (IQR 47.5–49.5 °C) in headache free participants and 48.2 °C (46.6–49.5 °C) in controls, (*P* = 0.43).

**Table 1:** Pain sensitivity

	Headache free (***n*** = 47)	Controls (***n*** = 52)	***P***-value
**Quantitative sensory testing**			
Cold pain threshold hand (°C)	1.1 (0.0–9.0)	1.9 (0.0–7.2)	0.81
Cold pain threshold forehead (°C)	4.6 (0.0–8.4)	5.1 (0.9–9.7)	0.38
Heat pain threshold hand (°C)	49.1 (46.8–50.4)	48.8 (46.8–50.2)	0.49
Heat pain threshold forehead (°C)	48.3 (47.5–49.5)	48.2 (46.6–49.5)	0.43
**Pericranial tenderness**			
Total tenderness score	9.6 (7.0–13.0)	10.4 (7.8–13.9)	0.70
Cephalic tenderness score	6.0 (4.4–7.9)	6.4 (4.8–8.4)	0.72
Neck tenderness score	3.7 (2.6–5.1)	4.1 (2.9–5.4)	0.67
**Cold pressor test**			
Verbal rating score	5.8 (5.1–6.4)	5.4 (4.8–6.1)	0.45
Time (min)	10.0 (4.8–10.0)	10.0 (3.8–10.0)	0.75

Normally distributed data presented as mean values with 95% confidence intervals and non-normally distributed data as median values with interquartile range. Differences between the two groups were assessed with the unpaired Student’s t-test or Mann-Whitney U test as appropriate.

### Pericranial tenderness

**Fig. 2:**
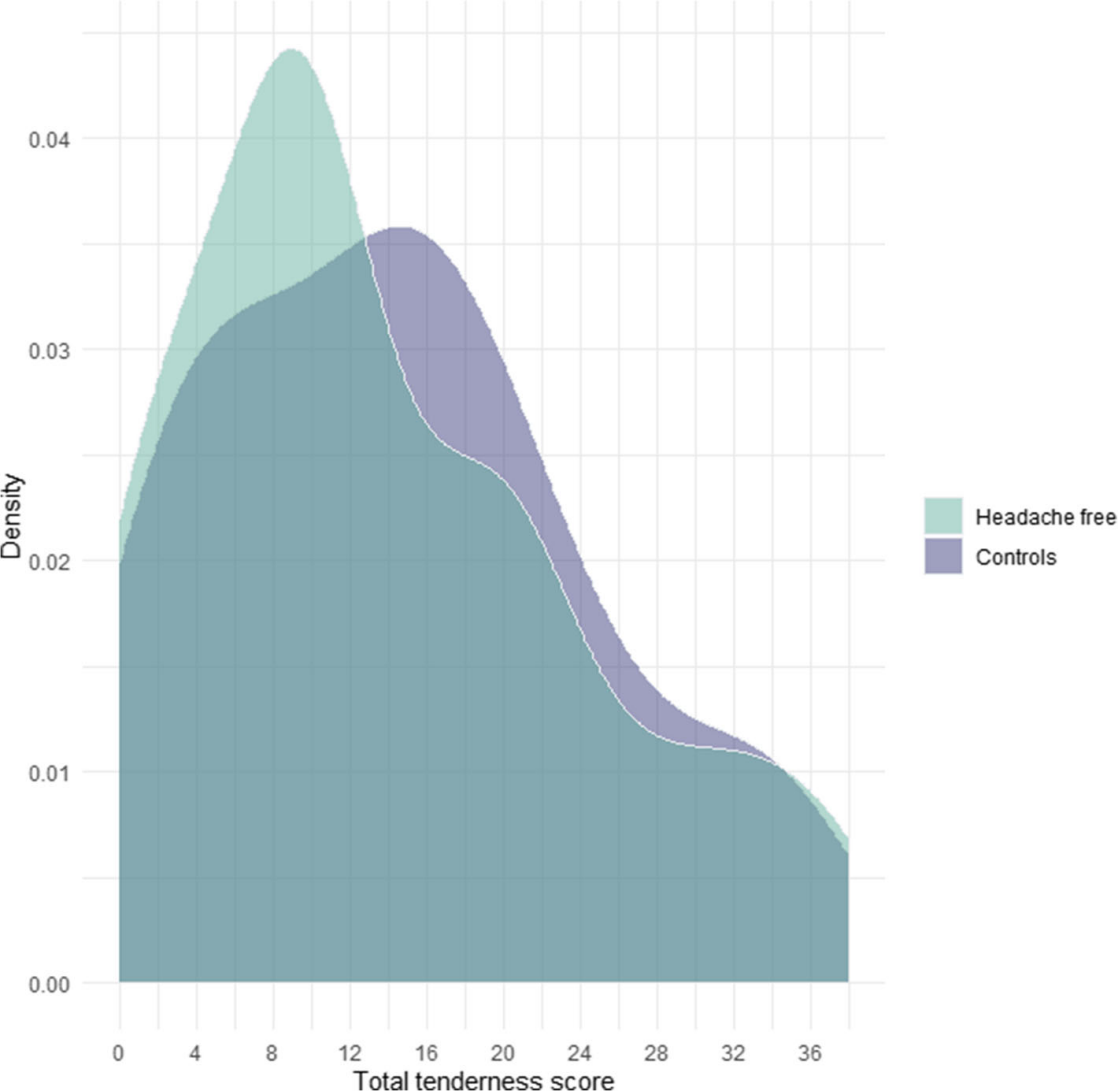
Distribution of total tenderness score. Comparison of the distribution of total tenderness score in headache free participants and controls

There was no difference in total tenderness score (TTS) between headache free participants and controls with a mean TTS of 9.6 (95% CI 7.0–13.0) and 10.4 (95% CI 7.8–13.9) respectively, (*P* = 0.70). Distribution of TTS in headache-free participants and controls are shown in Fig. 2. There was no difference in cephalic or neck tenderness score between the two groups. Mean cephalic tenderness score was 6.0 (95% CI 4.4–7.9) in headache free participants and 6.4 (95% CI 4.8–8.4) in controls, (*P* = 0.72). Mean neck tenderness score was 3.7 (95% CI 2.6–5.1) in headache free participants and 4.1 (95% CI 2.9–5.4) in controls, (*P* = 0.67).

### Cold pressor test

**Fig. 3:**
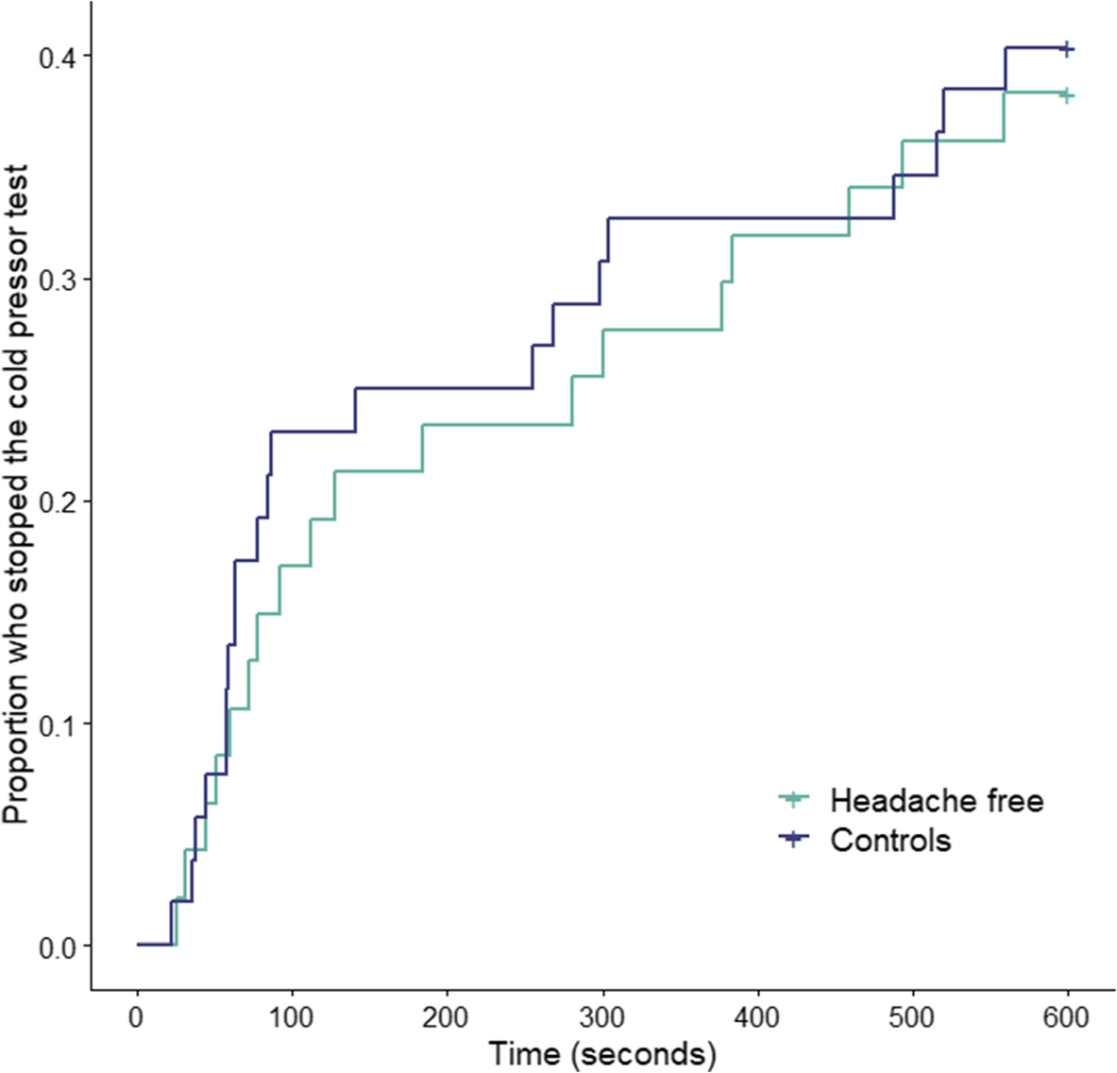
Pain tolerance. Proportion of headache free participants and controls who stopped the cold pressor test over time

There was no difference in pain tolerance between the two groups, there was a median pain tolerance time of 10.0 min (95% CI 4.8–10.0) in headache free participants and 10.0 min (95% 3.8–10.0) in controls, (*P* = 0.75). Kaplan-Meier curves of pain tolerance are shown in Fig. 3. There was no difference in survival time between groups, (*P* = 0.79). There was no difference in pain ratings after cold pressor test between groups with a mean VRS of 5.8 (95% CI 5.1–6.4) in headache free participants and 5.4 (95% CI 4.8–6.1) in controls, (*P* = 0.45).

## Discussion

Using a validated and standardized quantitative sensory testing (QST) protocol, total tenderness score (TTS) and cold pressor test, this observer blinded nested case-control study demonstrated that men who have never experienced a headache do not have a lower pain sensitivity when compared with controls.

### Quantitative sensory testing

There was no difference in pain detection threshold between headache free participants and controls in the QST parameters on neither hand nor forehead. Cold pain threshold (CPT) and heat pain threshold (HPT) reflect the function of the thinly myelinated A-delta fibers and unmyelinated C-fibers. The standard QST protocol includes 13 individual parameters for a complete assessment of the function of the somatosensory nervous system. In the present study we only used two of the 13 individual parameters. It is therefore possible that a difference in one of the other parameters, could be present. However, if there was a general difference between the two groups, we would expect to see a change even in the two parameters we tested. Supporting our results, Jensen et al. tested pressure pain threshold in people who had never experienced a headache and found no difference compared to the general population [19].

### Pericranial tenderness

We did not find a lower pericranial muscle tenderness measured by TTS in headache free participants compared with controls. Headache free participants reported a mean TTS of 9.6 in the present study. This pericranial tenderness is higher than we would have expected from other studies with TTS on headache free participants [19, 39]. In a study of pericranial tenderness in headache patients compared with headache free participants, they included 25 headache free participants (9 men and 16 women) and found no pericranial tenderness in any of the headache free participants [39]. However, this study was not observer blinded and the pressure exerted was just described as “mild”, not necessarily comparable to the present study with a manual palpation of 80 kPa. In a population-based study of muscle tenderness, the authors showed a significantly lower modified TTS in a sample of 26 headache free male participants compared with a general population sample of 383 male participants without a primary headache diagnosis, except infrequent tension type headache [19]. The study calculated TTS based on 14 pairs of muscles, compared to only 8 pairs of muscles in the present study, and found a mean TTS in headache free male participants of 5 (95% CI 2–8). The high pericranial tenderness in the present study could be due to differences in the classification of “never headache”. However, the prevalence of never headache in the population-based study of Jensen et al. matches the prevalence of headache free participants in the Danish blood donor study (DBDS) cohort, indicating that the studies capture the same part of the population. The different results between the present study and the two previous studies might also be due to statistical power as sample size differed with 47 headache free participants in our study, compared to 25 and 26 in the two earlier studies [19, 39].

Studies have found an increased pain sensitivity in people with tension type headache compared with healthy controls after a stressful muscle stimulus like the tooth clenching task [40, 41]. It is possible that there is a difference in pain sensitivity between headache free participants and controls, if they experience a stressful muscle stimulus before quantitative sensory testing and total tenderness score.

### Cold pressor test

Pain tolerance and pain rating during the cold pressor test were similar in headache free participants and controls. Pain tolerance was higher than in previous studies examining healthy volunteers [42, 43]. This could be due to our sample, as both male sex and increasing age are associated with a higher pain tolerance [44]. It could also be explained by the fact that we did not use a circulation system. If water is not circulated, a warmer microenvironment around the hand can occur if it is kept still. But as headache free participants and controls were examined the same way, this should not affect the difference between the two groups.

### Strength and limitations

To reduce the risk of observer bias, several steps were taken. The observer was blinded for the case-control status of the study participants. A validated standardized QST protocol was used and the observer was trained with pressure-controlled palpation to estimate total tenderness score. This is a validated method shown to increase the reliability and reproducibility of manual palpation [36, 37]. Data were collected and analyzed by the observer, while she was still blinded. Cases and controls were examined in a random order to minimize the risk of time-related bias.

There is no validated questionnaire to identify persons who have never experienced a headache. To minimize misclassification bias, participants answered the question on headache when they were included in the DBDS cohort, and again when they entered the present study. The question on headache was designed to include all types of headaches, both the primary headache disorders and symptomatic headaches like traumatic headache and headache related to alcohol consumption. However, we have not examined how the question where understood in the present sample. We hope to examine the selection question on headache freedom in the future.

We did not include participants with any type of chronic pain. On the study day participants had to have been without any headache for at least 48 h. However, we did not ask participants about any other type of acute pain prior to the study day, and this could create bias in our results. We did not collect information on headache through headache diaries during the study. Pain threshold can vary with migraine phase in people with migraine, giving rise to a possible bias in studies on pain sensitivity in migraine patients [45]. As the controls in the present study were a random sample of male blood donors, only 5 (10%) controls had episodic migraine, and this minimizes the risk that changes in pain threshold with migraine phase are affecting our results. Both cases and controls have been recruited from the same background population of Danish blood donors. This cohort differs slightly from the general population [29]. We have only examined pain perception in headache free men, and we cannot be sure that our findings can be translated to women who have never experienced a headache. We selected men only because there were many more headache free men and to eliminate sex related variability.

## Conclusion

Our study clearly shows that freedom from headache is not caused by a lower general pain sensitivity, examined by cold and heat pain detection threshold, pericranial muscle tenderness and pain tolerance. Our results support the hypothesis that headache is caused by specific mechanisms present in the primary headache disorders, rather than a higher general sensitivity to painful stimuli.

## Data Availability

The datasets used and analyzed during the current study are available from the corresponding author on reasonable request.

## Abbreviations

CPT: Cold pain threshold
DBDS: Danish blood donor study
HPT: Heat pain threshold
MAP: Mean arterial pressure
QST: Quantitative sensory testing
TTS: Total tenderness score
VRS: Verbal rating scale
